# Testing the efficacy of three informational interventions for reducing misperceptions of the Black–White wealth gap

**DOI:** 10.1073/pnas.2108875118

**Published:** 2021-09-13

**Authors:** Bennett Callaghan, Leilah Harouni, Cydney H. Dupree, Michael W. Kraus, Jennifer A. Richeson

**Affiliations:** ^a^Stone Center on Socio-Economic Inequality, City University of New York, New York, NY 10016;; ^b^School of Management, Yale University, New Haven, CT 06511;; ^c^Department of Psychology, Yale University, New Haven, CT 06511;; ^d^Institution for Social and Policy Studies, Yale University, New Haven, CT 06511

**Keywords:** social psychology, racism, economic inequality, social cognition

## Abstract

Americans remain unaware of the magnitude of economic inequality in the nation and the degree to which it is patterned by race. We exposed a community sample of respondents to one of three interventions designed to promote a more realistic understanding of the Black–White wealth gap. The interventions conformed to recommendations in messaging about racial inequality drawn from the social sciences yet differed in how they highlighted data-based trends in Black–White wealth inequality, a single personal narrative, or both. Data interventions were more effective than the narrative in both shifting how people talk about racial wealth inequality—eliciting less speech about personal achievement—and, critically, lowering estimates of Black–White wealth equality for at least 18 mo following baseline, which aligned more with federal estimates of the Black–White wealth gap. Findings from this study highlight how data, along with current recommendations in the social sciences, can be leveraged to promote more accurate understandings of the magnitude of racial inequality in society, laying the necessary groundwork for messaging about equity-enhancing policy.

## Significance

An intervention study exposed a US community sample to messages about Black–White racial inequality. Interventions including data bearing on Black–White wealth inequality elicited higher estimates of that inequality that persisted for at least 18 mo, aligning with federal data measuring the Black–White wealth gap. The data interventions also increased acknowledgment of White Americans’ structural advantage and reduced beliefs in personal achievement as the remedy for racial inequality. In contrast, a narrative-based intervention, including information on a single Black family contending with racial inequality, did not shift inequality estimates or change respondents’ explanations. This study suggests how social science data can be used to create more realistic perceptions of racial inequality—a prerequisite to enacting equity-enhancing policy.

In a recent study, a nationally representative sample of survey respondents estimated that, on average, Black families had $90 US in wealth relative to the $100 of US White families in the United States (1, 2). The mean estimates generated by respondents in this and other studies were between 40 and 80 percentage points higher for Black family wealth than median statistics collected using the federal government’s Survey of Consumer Finances (SCF), where at the time of this writing, Black families hold $12.81 US in wealth relative to White families (3). The magnitude of this misperception is vast (4, 5). Reducing this perceptual gap, with the larger aim of reducing racial inequality through policy, is the focus of this research.

In this study, we sought to better understand how to promote more realistic perceptions of racial inequality in the United States. We conducted a laboratory-based intervention on people’s estimates of the Black–White wealth gap—an inequality that Americans, on average, are shockingly ignorant of (1, 2). We then followed respondents who completed the intervention for up to 18 mo to determine if any changes in understandings of the wealth gap would persist. Our central prediction was that information communicating the magnitude of the Black–White wealth gap with data, versus exposure to a narrative featuring the hardships and lived experiences of a single family contending with obstacles that stem from the Black–White wealth gap, would be necessary to increase the perceived magnitude of Black–White wealth inequality in society.

We focus on wealth inequality in this study because it is the single most consequential economic indicator of the ability to absorb unanticipated financial shocks (1). The process for how Americans develop and sustain such a large gap between perception of the Black–White wealth gap and its reality is multifaceted. One barrier to a more accurate understanding of racial wealth inequality is a failure to reckon with the specific relationship between wealth accrual and societal racism, both past and present (6). The capacity to accrue wealth has been unequal for Black and White Americans since before the founding of the United States (when Black Americans constituted wealth) to the recent past, when policies like redlining and the GI Bill largely excluded Black Americans from their wealth-accruing benefits (6–8). Contemporary practices also create racially unequal capacities for Black and White Americans to accrue wealth. For instance, Black Americans need to take on significantly more student loan debt (8), are more likely to be incarcerated, earn lower wages, and live in neighborhoods with lower property values relative to Whites (8, 10).

The magnitude of Black–White wealth inequality can be startling for many people who learn of it for the first time, and yet many never learn about it at all. Some of the reasons for this lack of learning include the aversion to numeracy that leads people to avoid population statistics (11). A second barrier is misinformation about the Black–White wealth gap propagated by prevailing narratives about the exceptional and meritocratic nature of American life (12, 13). The narrative of racial progress is one such narrative that shapes our understanding of the Black–White wealth gap (14, 15). This narrative minimizes contemporary societal racial inequality, preferring instead to highlight the capacity for individual achievement regardless of race and to consider racial inequality as something that is rapidly, and perhaps naturally, decreasing over time (16, 17). As long as this narrative governs perceptions of societal racial equity, the magnitude of the Black–White wealth gap will remain an inconvenient truth that must be ignored.

Evidence suggests that people adhere to the narrative of racial progress, and that motivational processes supporting that narrative drive misperceptions of racial inequality in society (18–20). For instance, belief in a just world predicts the extent that Americans underestimate racial inequality (5). In more recent experimental work, efforts to incentivize accuracy through monetary compensation were successful in eliciting more accurate perceptions of racial wealth inequality (4), suggesting that these misperceptions are, at least in part, motivated by a desire to uphold beliefs in contemporary racial equality (21). In the following section, we compare the ability of different messaging strategies, based on personal narratives or data, to elicit more realistic perceptions of the Black–White wealth gap.

## Narratives, Data, and Realistic Perceptions of the Black–White Wealth

Social scientists have examined how to discuss racial inequality in ways that engender a more accurate understanding of its persistence and magnitude for at least a century (22). A central barrier to effective communication about racial inequality is the fact that accurate information often directly contradicts narratives of racial progress. As a result, such information is likely to trigger reactance rather than persuasion (2). Providing accurate information about the magnitude of racial inequality can trigger fear and stereotypes designed to justify it (23, 24). For example, when White people were exposed to prison population information including more Black Americans, they became significantly more fearful of crime and more punitive in their policy choices (23).

One approach to messaging involves the use of narratives that highlight the challenges that everyday people of color face because of racial inequality. Such a messaging strategy is likely to be effective because it makes the victims of racial inequality identifiable (25, 26), which activates compassionate responses to suffering and associated prosocial behaviors (27, 28). Such narratives can also provide information about how broad trends in racial inequality are experienced by individuals and thus may fill in the gaps of knowledge, among some Americans, that stem from living in a segregated society (7, 8). Given this analysis, we exposed a subset of our respondents to such a condition, providing a narrative of the lived experience of racial economic inequality from the perspective of a single Black American family (29).

While ample evidence indicates that narratives can shift people’s beliefs about racial inequality, messages that present concrete data may be necessary to penetrate what appears to be a profound ignorance of the Black–White wealth gap. In particular, the magnitude of the Black–White wealth gap and its associated misperceptions suggest that more detailed data on its magnitude might be needed to help respondents fundamentally recalibrate what they believe to be true (2, 4). For instance, general reminders, without data, of the persistence of structural racial inequality do not increase the accuracy of estimates of the Black–White wealth gap relative to a control condition without these reminders (30).

An approach to messaging about racial inequality that includes specific, relevant data are also well positioned to focus attention on not only the magnitude of the inequality but also on its structural determinants and correlates [i.e., the inequalities in education, healthcare, incarceration, and housing policy that maintain and reproduce racial wealth inequality (31, 32)]. Preliminary research suggests that data that attends to societal structures that produce inequality (e.g., educational funding gaps) might outperform personal narratives in shifting people’s beliefs: Personal narrative, versus data-based, presentations of inequality focus people on individual-level, typically achievement-based, paths to success (e.g., success based in individual talent and effort) and obscure the structural factors that create societal inequalities (e.g., laws and policy) by impeding (or facilitating) individual and/or group successes (33–35).

We reasoned that a data-based approach to messaging about the Black–White wealth gap would promote more realistic perceptions of that gap because it focuses on the magnitude and structural causes of racial economic inequality. In contrast, we expected a narrative approach to be less effective because it focuses respondents on individual efforts to overcome racial inequality, rather than on the societal structures that promote it. Thus, our primary hypothesis was that data-based messaging would be more likely than narrative-based messaging to elicit larger estimates of the Black–White wealth gap.

## The Present Research

In the present research, we tested the effectiveness of three interventions that used narrative- or data-based messages about the Black–White wealth gap. In the narrative condition, we highlight Black–White racial inequality in wealth, access to healthcare, housing, and prospects for economic mobility through the experiences of one family. In the data condition, respondents were informed about racial inequality with data, rather than a narrative, focusing on the structural nature of the Black–White wealth gap as well as gaps in education funding, housing, health care, and economic mobility. Importantly, the data condition directly informed people about the actual Black–White wealth gap in 2016 [SCF (31)]. The third condition presented the information in both the narrative and data conditions. This combined condition then provided both data on Black–White gaps in wealth and in other domains but also provided the personal narrative of the single Black family.

In our intervention, we also included the optimal conditions for persuasive messaging on the topic of racial inequality. To accomplish this, each intervention contained the following: 1) Incorporating insights from research on deep canvassing, we employed a nonjudgmental listening session in each intervention condition, given that such listening to voters’ opinions and personal experiences on an issue can effectively reduce transphobia and durably shift attitudes toward greater equity in the domain of immigration policy (36–38); 2) Aligning with research on race and class political messaging dynamics, our intervention conditions directly confronted racism as a problem and highlighted racial justice as a prerequisite to societal prosperity (39); and 3) We appealed to values that are commonly shared by all Americans, emphasizing the American Dream at the start of all interventions, to promote cross-group goals and reduce intergroup conflict (40–43).

As noted previously, we tracked the durability of any changes in perceptions of the Black–White wealth gap due to the interventions for up to 18 mo, beginning in April 2019 and concluding by November 2020. One concern about attempts to promote more realistic perceptions of the Black–White wealth gap is that such attempts will only have momentary effects (44). Because both exposure to and motivation for advancing narratives of racial progress are high in the United States (45, 46), any attempts to inform about the Black–White wealth gap are inevitably likely to run into counter examples and even targeted misinformation, as respondents go about their everyday lives, that will gradually push the conflicting information from our intervention to the periphery.

## Results

### Realistic Perceptions of the Black–White Wealth Gap.

**Fig. 1.**
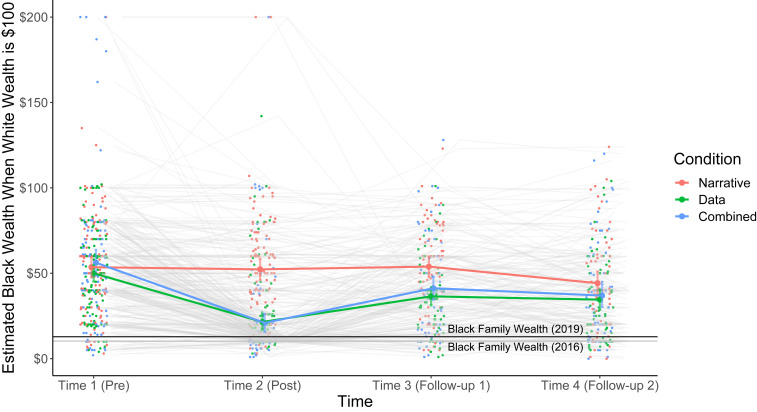
Estimates of Black wealth when White wealth is $100 US across our four time points for our three (narrative, data, and combined) intervention conditions. Raw data, means, and 95% CIs are displayed for each time point as a function of intervention condition. For reference, the estimated median Black–White wealth gap calculated based on data from the SCF in 2019 (black line) and 2016 (gray line) are plotted as horizonal lines.

Our central prediction was that data-based messages describing the magnitude of racial economic inequality would be more effective than narrative messages in shifting perceptions of the Black–White wealth gap to be more consistent with estimates of the actual median wealth gap as measured by federal data collected either in 2016 or 2019. We used a 3 (intervention) × 4 (time) mixed ANOVA to examine these predictions. The analysis yielded a significant time effect *F*(3,471) = 38.806, *P* < 0.001, an intervention effect *F*(2,157) = 11.418, *P* < 0.001, and a significant time by intervention interaction effect *F*(6,471) = 7.131, *P* < 0.001. To further explore this interaction, we examined comparisons between estimates produced at time 1, prior to the intervention, and subsequent time points for each of the three conditions (Fig. 1). Data were plotted using raincloud plots in R-Studio (47).

Importantly, no condition differences emerged at time 1 (*ts* < 1.40), but as predicted, both the data and combined conditions yielded significantly lower estimates of Black–White wealth equality at time 2 (data: *t*(111) = 11.001, *P <* 0.001, *d* = 0.98; combined: *t*(110) = 10.404, *P* < 0.001, *d* = 0.83) directly after the manipulation. The reduction in these estimates did not completely decay across time, persisting to time 3 (data: *t*(69) = 4.359, *P* < 0.001, *d* = 0.58; combined: *t*(65) = 3.214, *P* = 0.002, *d* = 0.40) and even to time 4 (data: *t*(66) = 5.136, *P* < 0.001, *d* = 0.67; combined: *t*(70) = 4.027, *P* < 0.001, *d* = 0.39) a full 18 mo after the start of the study. Though lower estimates compared to time 1 persisted across the 18 mo of the study, these estimates rose significantly from time 2 to time 3 (data: *t*(69) = −5.166, *P* < 0.00, *d* = 0.54; combined: *t*(65) = −6.189, *P* < 0.001, *d* = 0.57).

It is especially notable that the narrative condition, which focused on an identifiable family but did not highlight structural racial inequality (48) or directly inform on the Black–White wealth gap, did not yield significantly lower estimates of Black–White wealth equality at time 2 *t*(108) = 0.647, *P* = 0.519, *d* = 0.22 or time 3 *t*(72) = 0.722, *P* = 0.473, *d* = 0.12. Moreover, respondents in this condition were significantly higher in their estimates of Black–White wealth equality than either the data or combined conditions at time 2 (data: *t*(219) = 8.03, *P* < 0.001, *d* = 1.34; combined: *t*(219) = 7.59, *P* < 0.001, *d* = 1.28) and time 3 (data: *t*(142) = 4.30, *P* < 0.001, *d* = 0.89; combined: *t*(140) = 2.78, *P* = 0.006, *d* = 0.57). Estimates in the data and combined conditions were not significantly different at any time point (*ts* < 1.40).

Estimates of the Black–White wealth gap in the narrative condition did become lower at time 4 compared with time 1 *t*(65) = 3.298, *P* = 0.002, *d* = 0.36. Although this difference was not anticipated, it likely arose due to increased general societal awareness of racial disparities due to the COVID-19 pandemic (49) or police-perpetrated racial violence during the time in which those estimates were solicited (50). Despite this change among those in the narrative condition, their estimates of racial wealth equality continued to be significantly higher compared to the data condition *t*(131) = 2.12, *P* = 0.036, *d* = 0.37, with the combined condition falling in between (versus the narrative condition: *t*(137) = 1.50, *P* = 0.14, *d* = 0.26; versus the data condition: *t*(138) = 0.521, *P* = 0.600, *d* = 0.09). This observed decrease in the narrative condition estimates at time 4 echoes the central prediction and finding of this work—providing information regarding the magnitude of, and structural conditions that create and sustain, racial inequality in society can shift perceptions of that inequality in alignment with federal data.

Importantly, though we do see significant and sustained higher respondent estimates of the Black–White wealth gap following the data interventions, these estimates are still too optimistic regarding the actual degree of racial economic equality in contemporary society. Even at time 2, directly after respondents in the data and combined conditions were exposed to the current magnitude of the Black–White wealth gap, average estimates of racial wealth equality in the combined condition (where respondents showed the lowest estimates at time 2) were still over 10 percentage points higher than median racial wealth equality reported in the 2016 SCF *t*(111) = 3.965, *P* < 0.001, *d* = 0.31 and over 8 points higher than the median in the 2019 SCF *t*(111) = 3.027, *P* = 0.003, *d* = 0.23.

As Table 1 shows, the same pattern of results was robust to adjustments for race, gender, income, educational attainment, political ideology, and age. In a linear mixed model analysis where time was nested within respondents, we found a significant main effect of race with White people across conditions providing larger overestimates of Black–White wealth equality than people of color. We also found a main effect of intervention type, an effect of education such that those with lower educational attainment provided larger overestimates, an effect of political ideology such that those who were more conservative provided larger overestimates, no effect of gender, no effect of income, no effect of age, and a significant intervention by time interaction *F*(6,574) = 3.80, *P* = 0.001. As described in *SI Appendix*, there was no three-way interaction between race, the intervention, and time, suggesting that the data and combined conditions promoted more realistic perceptions of the Black–White wealth gap for both White respondents and respondents of color.

**Table 1.** Linear mixed model fixed effects with time nested within respondents where estimates of the Black–White wealth gap were predicted by the intervention, time, and their interaction, as well as race, gender, income, education, ideology, and age

	Estimate	SE	df	*t* value
(Intercept)	52.98	9.84	164.06	5.39*
Data intervention	−8.30	5.65	367.08	−1.47
Combined intervention	2.13	5.58	372.46	0.38
Time2	−4.80	4.47	438	−1.07
Time3	−3.32	4.47	438	−0.74
Time4	−12.36	4.47	438	−2.77*
Race	8.95	3.48	140	2.57*
Gender	−0.44	3.71	140	−0.12
Income	−0.03	0.78	140	−0.04
Education	−4.91	1.87	140	−2.62*
Ideology	4.63	1.25	140	3.70*
Age	0.19	0.14	140	1.34
Data × Time 2	−24.51	6.07	438	−4.04*
Combined × Time 2	−34.47	6.04	438	−5.71*
Data × Time 3	−12.07	6.07	438	−1.99*
Combined × Time 3	−16.19	6.04	438	−2.68*
Data × Time 4	−5.73	6.07	438	−0.94
Combined × Time 4	−7.37	6.04	438	−1.22

*P < 0.05.

### Speech and Attitudes about Achievement and Advantage.

One straightforward explanation for lower estimates of Black–White wealth equality in the data and combined interventions stems, of course, from the fact that data regarding the 2016 Black–White wealth gap were provided to respondents in those conditions but not in the narrative intervention. However, we did not expect this facet of the intervention to be the only reason for differential efficacy. We expected the data (and combined) intervention to be more effective than the narrative intervention in generating lower estimates of Black–White wealth equality for two additional reasons: 1) the data-based intervention centered information on the longstanding and persistent structural underpinnings of racial inequality in American society (35) and 2) the narrative condition centered information on individual efforts to overcome adversity. Thus, respondents in the narrative condition should be more likely to focus on personal achievement and striving and less on the roles of systems and structures shaped by law and policy as explanations for racial inequality compared with respondents in the data (and combined) condition.

Because this analysis was exploratory and because we observed no differences between the data and combined conditions across our outcome measures, we merged these conditions for this analysis and compared them against the narrative condition. We then examined achievement-related speech use during the nonjudgmental listening session to get a sense of how respondents talk about racial inequality following our intervention using the Linguistic Inquiry and Word Count (LIWC) achieve dictionary (51). We also examined respondents’ structural beliefs about racial inequality by examining responses to two survey questions about beliefs that White Americans have an unfair advantage in society, asked following the nonjudgmental listening session (35).

To examine the tendency to use achievement-related words as a function of our intervention, we conducted a linear regression analysis predicting use of achievement words with our intervention, race, gender, income, ideology, educational attainment, age, and other linguistic patterns around word count, using words of six letters or more, positive emotion, and negative emotion. Our experimental manipulation significantly predicted achievement-related words such that more of these words occurred in the narrative condition versus the other two conditions (see Table 2). In addition to the intervention type, age, use of longer words, and use of positive words were associated with use of achievement words.

**Table 2.** Linear regression analysis predicting use of achievement-related words

Variable	B	SE	*T*
(Constant)	0.020	0.44	0.05
Data/combined intervention	−0.26	0.12	−2.15*
Race	−0.05	0.06	−0.92
Gender	−0.20	0.12	−1.70+
Income	−0.01	0.03	−0.46
Ideology	0.00	0.04	0.05
Education	−0.09	0.06	−1.37
Age	0.01	0.01	2.47*
Six letter words	0.08	0.02	4.48*
Word count	−0.00	0.00	−0.99
Postive words	0.35	0.04	9.24*
Negative words	−0.13	0.09	−1.50

*P < 0.05; ^+^*P* < 0.10.

A qualitative examination of respondent speech reveals a similar pattern. When asked about equity-enhancing policy during the listening session, those in the narrative condition mentioned people overcoming racial inequality through their own achievements. One respondent suggested that the government create a program that “follows, like Black/Latinx youth, um, from an early age that helps them, um like stay in school and like give them whatever they might need to succeed.” Another respondent in the narrative condition reflected on their own lack of success and opportunities for their children: “the opportunities to be able to go to school. Um, I’m supportive with that, cause when I had the opportunity, I kind of blew if off.” A third respondent in the narrative condition focused on helping disadvantaged students “catch up:” “if you come from a disadvantaged family that you can sort of catch up in a sense for whatever, whatever catch up means for a four year old.”

In contrast, though all interventions ended by stating that policy change was necessary, respondents in the data and combined conditions were more likely to talk about reducing funding deficits through policy. Most notably, the word “redistribution” was mentioned five times in the policy portion of the interview and only among respondents in the data-based interventions. One respondent said, “I’m out fully for like the redistribution of like wealth.” And another remarked that in particular “redistribution of wealth in school districts? Um, I think they should all be funded equally or even for a school district should be funded more to make it equitable, um, not just equal.”

We also analyzed two questions assessing respondents’ prescriptive beliefs about how opportunities ought to be distributed in society and how much the government ought to do to address racial inequality, adapted from the General Social Survey (GSS), [e.g., “Do you think that White Americans have more opportunities than they should, that Black Americans have more opportunities than they should, or that opportunities are about equal between racial groups?” (35)]. In this linear regression analysis, we predicted perceptions of White structural advantage at time 2, with the same scores at time 1, the data/combined intervention, and control variables that included race, gender, income, ideology, educational attainment, and age (Table 3). As Table 3 shows, respondents exposed to the data/combined intervention tended to endorse higher levels of White structural advantage in society relative to those in the narrative condition. Time-1 responses of White structural advantage were a significant predictor of time-2 responses, as was ideology, with more liberal respondents tending to report greater White structural advantage. No other effects were significant.

**Table 3.** Linear regression analysis predicting perceptions of White structural advantage at time 2

Variable	B	SE	*T*
(Constant)	2.10	0.33	6.43*
Data/combined intervention	0.18	0.08	2.27*
White structural advantage (time 1)	0.74	0.04	18.36*
Race	0.07	0.04	1.80+
Gender	−0.09	0.08	−1.19
Income	0.00	0.02	0.18
Ideology	−0.13	0.03	−4.07*
Education	−0.05	0.04	−1.21
Age	0.00	0.00	0.04

*P < 0.05; ^+^*P* < 0.10.

### Equity-Enhancing Policy Support.

We also explored the influence of our interventions on two equity-enhancing policies—support for a baby bonds program to reduce the racial wealth gap and a federal job guarantee program to provide working adults an option to work for the federal government. A 3 (time) × 3 (intervention) ANOVA found no effects for the federal job guarantee program or baby bonds for time, condition, or their interaction *Fs* < 2.29, *ps* > 0.10. Although our intervention analysis did not show evidence of impact on policy support, exploratory correlational analyses do suggest that some attitudes and beliefs that shifted during the intervention were associated with more support for baby bonds and the federal job guarantee program. For instance, reporting greater White structural advantage at time 2 was positively associated with support for both baby bonds *r*(302) = 0.34, *P* < 0.001 and the federal job guarantee *r*(302) = 0.28, *P* < 0.001. As well, when relaxing statistical significance criteria to *P* < 0.10, lower estimates of the Black–White wealth gap at time 2 were associated with greater support for baby bonds *r*(302) = −0.11, *P* = 0.056 but not for the federal job program *r*(302) = −0.02, *P* = 0.683.

## Discussion

Ignorance and misinformation are central components of the practice and maintenance of racial inequality in American society. Today, efforts to misinform the public about the legitimacy of the voting rights of Americans in major cities and counties, disproportionately inhabited by communities of color, threaten the democratic principles of the US government (52). In the past, similar patterns of misinformation have contributed to racial disparities in other domains of life (7, 53). These conditions highlight the importance of a public that holds a realistic perception of American society, including the existence of racial inequality and its magnitude. Realistic understandings of society could also serve as powerful buffers against misinformation campaigns that cast the country as free from the very forces that threaten to undermine its institutions and principles (54).

In this study, we tested three interventions to combat misinformation and educate Americans about the persistence of Black–White wealth inequality in society. Overall, interventions including data lowered perceptions of Black–White wealth equality to levels that were more consistent with federal data. Moreover, these perceptions persisted up to 18 mo after the intervention and did so for White respondents and respondents of color. Although the data-based intervention resulted in larger estimates of the Black–White wealth gap, this effect significantly decayed across the 18 mo of the study. Moreover, even just after informing respondents (in the data and combined conditions) of federal data on the racial wealth gap, they still significantly overestimated it. These latter findings suggest a clear, yet peculiar, commitment to the misperception of racial economic equality. Narratives of societal racial progress are difficult for even veridical information presented in a single laboratory session to penetrate (16, 17, 30).

Nevertheless, this work offers promising evidence that an informational intervention can successfully shift respondent perceptions of racial economic equality, at least when communicating about racial inequality with data and their structural underpinnings, perhaps supported by nonjudgmental listening (38). As well, given the rising estimates of Black–White wealth equality across time, more comprehensive intervention approaches must be engaged at a far-wider scale to permanently shape understanding of racial inequality in America’s past and present (55, 56). Though the exact dosage of such an intervention is a topic of future research, we suggest that small and continuous messages that could be deployed as part of public educational initiatives about racial justice would be preferable to single-shot interventions like the one conducted in this research. Unlike moments of widespread awakening to racial justice concerns that tend to be short lived and temporary (49,50), such continuous intervention would be more effective for debunking the misinformation presented as part of highly salient American narratives of racial progress (5).

It is especially noteworthy that respondents in the data and combined conditions referred to personal achievement less and endorsed structural advantages held by White Americans more in their speech behavior, directly following the intervention, compared with respondents in the personal narrative condition. This speech finding is important because cross-race encounters are both a significant cause of stress for communities of color (57, 58), yet also a potential context for prejudice reduction (57, 59). Interventions like this one can reduce the reliance on achievement-based narratives that, intentionally or otherwise, paper over structural racial inequalities. Such interventions have the potential to improve cross-race contact by focusing conversations about race and racism on the structural determinants of racial inequality and should be a topic of future research (35, 60).

Importantly, despite changing people’s beliefs about Black–White wealth inequality and how they spoke about that inequality, we did not observe corresponding shifts in economic policy support, a pattern aligned with ongoing research in this area (32). The connection between accurate conceptions of racial wealth inequality and policy is one in need of additional inquiry. Such research could consider how respondents understand the links between the structural patterns of Black–White wealth inequality, such as the ones we described in the intervention, and how specific policies might disrupt these structural practices so as to reduce racial inequality. In the case of support for the baby bonds program, for instance, it is possible that respondents would need additional information about the cost of such a policy, how much Black wealth would increase over time as a function of the creation of these federal bond accounts, and how much such increases would decrease wealth inequity between Black and White families (61). While clearly not a sufficient condition for engendering policy support, then, increasing the accuracy with which people perceive racial wealth equality is likely to be a necessary condition for understanding and engaging in any efforts to reduce the racial wealth gap. The current intervention study offers insight into at least one avenue through which to promote more realistic perceptions of the Black–White wealth gap—a profoundly misunderstood, yet significant, pattern of racial inequality in society.

## Materials and Methods

### Study Overview.

In this intervention study, respondents answered a brief survey about their perceptions of society. Respondents then came to our laboratory to receive one of three randomly assigned experimental interventions aimed at promoting more realistic perceptions of the Black–White wealth gap, engaged in a listening session where they expressed their views of and reactions to the material presented about racial wealth inequality and then provided their estimates of this gap. Following the laboratory session, respondents provided these estimates via email, two additional times over a period of up to 18 mo. Data and materials for the study, as well as preregistrations for analyses for the initial study and follow-up, can be found online (https://osf.io/e9jky/).

### Respondent Sample.

Respondents were recruited through the Yale Behavioral Laboratory’s community subject pool as part of a study of attitudes about society. The experimental procedure was approved by the institutional review board at Yale University, and all respondents consented to participate in the study. This subject pool was used to obtain a sample of the surrounding community of New Haven, CT, United States, with the specific goal of overrepresenting community members from racial backgrounds that are historically underrepresented in the social sciences (62). An email with a link to an initial invitation to participate in the experiment was sent out to the list of community respondents maintained by the laboratory. All respondents were over the age of 18. This method yielded an initial sample of 339 respondents who provided data at time 1 (preintervention) and time 2 (postintervention), 223 (65.8%) of which responded to follow-up measures at time 3 (follow-up #1), and 206 (92.4%) of those respondents responded at time 4 (follow-up #2). Respondents who completed time-3 measures did so, on average, 96.5 (SD *=* 27.92) d after the intervention. For time-4 measures, responses occurred, on average, 341.36 (SD = 13.66) d after time 3 and 436.67 (*SD* = 30.53) d after the intervention, with the maximum lag between intervention and time 4 being 524 d.

No exclusions were made, except in cases of missing data. When analyzing responses for missing data, we took two approaches. In the analyses reported here, we excluded cases listwise based on missing data. We also imputed responses using inverse probability weighting, and this analysis appears in the *SI Appendix* (63). In both cases, the conclusions reached from our analyses were identical. A binary logistic regression analysis on attrition rates, included in the *SI Appendix*, revealed no systematic variation as a function of demographic characteristics or general political attitudes, nor did it reveal differential attrition by intervention condition.

Respondents completed a battery of demographic items, including gender, age, educational attainment (1 = Less than high school, 2 = High school graduation or equivalent, 3 = Some college, 4 = College graduation, and 5 = Professional/postgraduate degree), income (measured in increments of $10,000; 1 = Under $15,000, 8 = Over $150,000), and political orientation (1 = Very Liberal, 7 = Very Conservative). Most respondents identified as women (*n* = 196), 135 identified as men, three identified as “non-binary or other,” and five did not disclose. The mean age for this sample ranged from 18 to 63 y old (M = 32.30; SD = 11.93). On average, respondents had completed some college or a college degree (M = 3.64, SD = 1.01) and made between $35,000 and $50,000 per y (M = 4.47, SD = 2.24). Respondents were, on average, slightly more politically liberal (M = 3.09, SD = 1.43). Based on responses to survey questions and during in-laboratory interviews, respondents were categorized as White (*n* = 140) or persons of color (*n* = 183), and among them were 39 respondents identified as Black Americans.

### Procedure and Intervention.

Respondents were recruited via online and publicly posted advertisements in April of 2019 to take part in a combined survey and laboratory study across four time points: a preintervention email survey (time 1), a postintervention survey (time 2), and two follow-up email surveys (times 3 and 4). The intervention session occurred in the laboratory, immediately before the time-2 survey. Perceptions of racial economic inequality were assessed at each of the time points to assess changes, by condition and over time. Table 4 presents a timeline of the study. Respondents were first provided with a definition of wealth and income as in prior research (64) and then proceeded to their estimates of Black–White wealth inequality. Respondents also provided estimates for general wealth inequality.

**Table 4.** Timeline of events, including administration of survey measures, for the study

Event	Method	Date
Pre-Survey	Email	April 2019
Intervention	Laboratory	April 2019
Postsurvey	Laboratory	April 2019
Follow-up #1	Email	October 2019
Follow-up #2	Email	September 2020

At the laboratory, respondents were randomly exposed to one of the three video interventions: the narrative, data, or combined conditions. Each of the three interventions consisted of a video that discussed Black–White racial inequality (https://osf.io/e9jky/). Each intervention video began with a brief narrated introduction of a concept widely endorsed by people across the United States, the concept of the American Dream (13). Next, the narrator questions whether the opportunities and prosperity that are promised as rewards for hard work and effort, which are central to the American Dream, are available to all Americans.

Following this introduction, the video diverges into the information discussed and described in the three intervention conditions: The narrative condition provided context for racial inequalities in society by relaying a personal story of struggle for educational opportunities. This condition was designed to personalize the experience of those adversely impacted by racial inequality and to portray an identifiable family facing such circumstances. The narrative condition relayed the true story of a Black high school student attempting to go to college while facing (9) structural conditions that make educational opportunities difficult to reach (29). These structural conditions include eviction and financial insecurity, rising healthcare costs for family members with disabilities, and family unemployment.

The data condition provided context for racial inequalities by relaying data describing the disproportionate structural barriers facing Black American families in America. The data included statistics on inequality in public education funding (65), home values caused by redlining (8), racial (and gender) differences in upward mobility (61), and most importantly, presented the 2016 current Black–White wealth gap. The structural causes (i.e., the role of current and past laws and policies) of each of these inequalities were described.

The combined condition provided the information from both the narrative and data interventions. At the end of each intervention, connections between the narrative and data were made to the American Dream, and each condition ends by asserting that policy changes to society that create more equal opportunities will promote shared prosperity. Both the narrative and data videos were around 3.5 min in length, whereas the combined video was a little over 5 min.

Immediately following the intervention, an experimenter told respondents they were interested in learning about their opinions and impressions of the video. At this time, video recording began. Respondents were asked to verbally answer the following four questions: 1) “The video that you watched described how opportunities are unequal for people. What is your family’s experience with the American Dream?” 2) “The video pointed out structural inequalities that disproportionately affect Black and Latinx Americans. What is your opinion on the size of the role and impact of race on opportunities in America?” 3) “What are some other factors not discussed in the video, that you think are relevant to how people take advantage of opportunities in America?” 4) “The video mentioned equity-enhancing policies that create a more even playing field. What is a government policy that you would support that would help make opportunities more equal?”

These questions were designed to afford respondents the opportunity to connect their own experiences to the information they learned in the interventions and to think about policy responses. We informed respondents that we were interested in learning about their impressions of the video and how the information presented was related to their experiences. As in prior research, experimenters were instructed to listen to responses in a way that was engaged, nonjudgmental, and without interruptions (38). Interview answers were transcribed and analyzed using LIWC software (51).

After this nonjudgmental listening session, respondents completed a postintervention survey (at time 2) that included the same measures at time 1 and two additional policy support questions, individual difference measures reported in *SI Appendix*, and demographic measures. Though respondents were unaware at time 2, two online follow-up questionnaires were sent to respondents to evaluate whether the interventions had a lasting effect on respondents’ perceptions of inequality. The time-3 survey was sent out nearly 2 mo after completion of the laboratory portion of the study and contained the same inequality estimates and policy support measures as at time 2. The same procedure applied to the second follow-up, which took place between September and November of the following year.

### Measures.

#### Perceptions of the Black–White wealth gap.

As the primary dependent variable of this study, respondents estimated the distribution of wealth between Black and White Americans. The item was based in prior research (5). Respondents were asked “For every $100 in wealth accumulated by an average White family, how much wealth has the average Black family accumulated?” (M_T1_ = 53.11, SD_T1_ = 33.1) and answered on a 0 to 200 slider scale where 100 represents equality. To examine accuracy, we compared the mean of these perceptions and federal median estimates of the Black–White wealth gap in 2016 ($10.29 US) and 2019 ($12.81 US) as reported by the SCF (3).

#### Postintervention discussion of racial inequality.

To assess what respondents thought following the intervention, we analyzed the content of their response to the postintervention questions using LIWC (51). Respondents answered the four postintervention questions in an average of 541.41 words (SD = 438.05). We were interested in how much respondents referred to achievement-based narratives of success directly following the interview. We used the “achieve” dictionary (e.g., ability, accomplish, progress, success, ambitious, attain, and achieve) from LIWC to conduct this analysis (M = 2.12, SD = 1.14). As controls, we also used LIWC to assess the total words, words per sentence (M = 18.24, SD = 5.01), words of six letters or more (M = 14.87, SD = 3.40), positive emotion words (M = 3.58, SD = 1.56), and negative emotion words (M = 0.83, SD = 0.67).

#### Beliefs about racial inequality.

The two questions from the GSS assessing White structural advantage were assessed on seven-point scales: “Do you think that White Americans have more opportunities than they should, that Black Americans have more opportunities than they should, or that opportunities are about equal between racial groups?” (1 = Black Americans have too much, 4 = Things are about equal, and 7 = White Americans have too much; M_T1_ = 5.56, SD_T1_ = 1.25) and “Do you think the government is doing too little, too much, or about the right amount to address economic inequalities between White and Black Americans?” (1 = Far too little, 4 = About the right amount, and 7 = Far too much). This item was reverse-coded such that higher values represent desires for the government to do more (M_T1_ = 5.67, SD_T1_ = 1.32). These questions were assessed at three time points throughout the experiment (times 1, 2, and 4).

#### Equity-enhancing policy.

Support for two equity-enhancing policies were assessed on a 1 (the government should not adopt a [baby bonds/federal jobs guarantee] program) to 7 (The government ought to adopt a [baby bonds/federal jobs guarantee] program) scale. The first was a “baby bonds” program, which was described as providing “need-based bank accounts for every child born in the United States” (66). The second was a federal job guarantee program which “provides a job to anyone willing to work” (67). These items were administered at times 2 to 4; respondents tended to express neutral or slightly favorably opinions of a baby bonds (M_T2_ = 4.35, SD_T2_ = 1.35) and federal job guarantee (M_T2_ = 5.07, SD_T2_ = 1.83) program. Individual difference measures and additional perceptions of inequality are reported in the supplementary materials, along with data on activities during the pandemic collected as part of a larger project.

## Supplementary Material

Supplementary File

## Data Availability

Survey data and materials data have been deposited in Open Science Framework (10.17605/OSF.IO/E9JKY).
