# Supplementary material for: Testing the efficacy of three informational interventions for reducing misperceptions of the Black–White wealth gap
Source: Proc Natl Acad Sci U S A. 2021 Sep 13;118(38):e2108875118. doi: 10.1073/pnas.2108875118 (PMC8463878; doi:10.1073/pnas.2108875118)
Supplement: Supplementary File [file pnas.2108875118.sapp.pdf]

Supplementary Materials for: Testing the efficacy of three informational interventions for reducing misperceptions of the Black-White wealth gap

Bennett Callaghan[1], Leilah Harouni[2], Cydney H. Dupree[2],

Michael W. Kraus[2,3]*, Jennifer A. Richeson[3,4]

[1]City University of New York, New York, NY 10016

[2]Yale University, School of Management, New Haven, CT 06511

[3]Yale University, Department of Psychology, New Haven, CT 06511

[4]Yale University, Institution for Social and Policy Studies, New Haven, CT 06511

*Correspondence concerning this article should be addressed to Michael W. Kraus (michael.kraus@yale.edu), Yale University, 165 Whitney Ave., New Haven, CT 06511.

Classification: Social Sciences; Psychological and Cognitive Sciences

Keywords: Social Psychology; Social Cognition; Racism; Economic Inequality

Supplementary Methods and Measures

Additional Study Procedure Details

Respondents received $5 each for completing surveys at time 1, 3, and 4 and an additional $15 for completing the laboratory visit. Respondents consented to video recording during the nonjudgmental listening portion of the study but were also allowed to withdraw consent after completing the session. Video recordings ended after respondents had a chance to answer our four interview questions. As respondents were escorted out of the laboratory, experimenters gave a brief debriefing and made sure that respondents were willing to be contacted again by the behavioral lab. At this time, respondents did not know they would be recontacted for this study.

For recruitment to our two follow up surveys, respondents received three emails before they were considered non-responsive. Following data analysis for our final follow-up survey, respondents received a thorough written debriefing about the study and a preliminary report detailing the results of the intervention and how their data would be summarized for future scientific reports. Respondents were encouraged to provide further commentary about the research at this time, and several did so.

Perceptions of inequality in other domains. We also asked respondents about current levels of Black-White income inequality using the same methodology. Respondents were asked, “For every $100 earned by an average white family, how much do you think is earned by an average black family?” (M_{T1} = 58.58, SD_{T1} = 27.9). Respondents were reminded that selecting 100 represents equality for this item and responded on the 0-200 slider scale.

We also measured perceptions of general wealth inequality based on prior research (62). Respondents were asked "If the US consisted of 100 individuals with 100 units of wealth, how much wealth do each of the following groups [top 20/the second 20/the middle 20/the fourth 20/and the bottom 20] have?" Respondents then allocated these 100 units of wealth among the quintiles (responses were forced to sum to 100). We used estimates of the wealth owned by the top 20% as a measure of general estimates of class-based wealth inequality (M_{T1} = 60.11, SD_{T1} = 26.23). As an additional measure of wage inequality, respondents were asked to estimate how much an average Chief Executive Officer (CEO) earns in income for every $1 the average unskilled worker earns, on a 0 to 600 scale (M_{T1} = 365.78, SD_{T1} = 177.32; 1).

Individual difference measures. For exploratory purposes as part of a larger study, we also assessed several beliefs about economic opportunities and society, as well as several individual difference measures. An 8-item scale assessed respondents' beliefs regarding how easy it is to change one's social class (2). Four statements asked respondents to indicate their agreement with a number of statements (e.g., "It is common for people who are motivated enough to go 'from rags to riches'"; 1 = *Strongly disagree,* 4 = *Neither agree nor disagree;* 7 = *Strongly agree*) and two asked respondents to indicate how easy or difficult it is to change one's social class (e.g., "These days, how easy is it to change one's social class?"; 1 = *Very hard,* 7 = *Very easy*). This scale formed a reliable index (α = 0.89; M_{T2} = 3.15, SD_{T2} = 1.00).

Social dominance orientation (SDO) assesses support for inequality among social groups and was measured using an 8-item scale ($SDO_{7(S)}$; 3; α = .84). Respondents rated their opposition or support for several ideas on 7-point scales (*1 = Strongly oppose*; 4 = *Neither oppose nor favor;* 7 = *Strongly favor*). Example items include, "Some groups of people are simply inferior to

other groups" and "We should do what we can to equalize conditions for different groups" (reverse-coded).

Supplementary Analyses

Intervention impact on estimates of inequality in other domains. Interestingly, though our manipulation did not include specific information on income disparities between White and Black Americans, respondents in the data and combined conditions applied what they learned to the Black-White income gap, as noted by significant time $F(3,471) = 40.840$, $p < .001$ and intervention main effects $F(2,157) = 12.396$, $p < .001$, that were qualified by a significant time by intervention interaction $F(6,471) = 8.530$, $p < .001$. When probing pairwise differences in time 1 a similar pattern emerged with respect to respondent estimates. Significant reductions emerged in estimates of the racial income equality at time 2 among respondents in the data $t(110) = 11.515$, $p < .001$) and combined ($t(110) = 9.074$, $p < .001$) conditions, as well as at time 3 (structural $t(69) = 4.030$, $p < .001$; combined $t(65) = 2.733$, $p = .008$), and time 4 (structural $t(66) = 5.093$, $p < .001$; combined $t(70) = 3.453$, $p = .001$)— a full 18 months after the start of the study. By contrast, estimates of the Black-White income gap made by respondents in the narrative condiiton did not differ between time 1 and time 2 ($t(108) = 1.122$, $p = 0.264$) or time 3 ($t(72) = 0.110$, $p = 0.912$). At time 4, as was the case for respondents' wealth equality estimates, estimates of Black-White income inequality among those in the narrative condition were larger at this time point, compared with time 1, $t(65) = 2.733$, $p = .008$.

We also examined both general wealth inequality and CEO pay, which respondents reported at three time points, as a function of our three intervention conditions. Unlike estimates of racial wealth equality, estimates of general wealth equality showed a significant effect of time $F(2,416) = 5.494$ $p = .004$, no effect of intervention $F(2,208) = 0.125$, $p = .883$, and no time by

intervention interaction $F(4,416) = 1.398$, $p = 0.234$. Estimates of CEO pay showed the same significant time effect $F(2,414) = 3.250$, $p = .040$, no effect of intervention $F(2,207) = 2.260$, $p = .107$, and no interaction between time and intervention condition $F(4,414) = 0.583$, $p = 0.675$. For both general wealth of the top quintile and CEO pay, estimates of inequality were larger at time 2 (quintile $t(334) = 4.587$, $p < .001$; CEO $t(334) = 4.092$, $p < .001$) than at time 1. For time 3, only wealth quintile estimates were still larger than time 1, $t(210) = 2.377$, $p = .018$. CEO wage estimates were not significantly different from time 1 estimates following the intervention $t(209) = 0.441$, $p = .660$. Overall, our intervention promoting more realistice perceptions of the racial wealth gap did not differentially shape general estimates of inequality in wealth or CEO pay.

Moderation by respondent race. We also explored whether respondent race moderated the effects of the intervention in a 2(race)X3(intervention)X4(time) ANOVA. Our results were similar to those reported without accounting for respondent race in that the significant time by intervention interaction emerged $F(6,568)=3.45$, $p=.002$, with no effect of time $F(3,568)=1.27$, $p=.285$ and a significant intervention effect $F(2,568)=22.96$, $p<.001$. Though respondent race did not interact with time $F(3,568)=0.73$, $p=.534$, or intervention $F(2,568)=1.34$, $p=.262$, a main effect of respondent race emerged $F(1,568) =16.75$, $p<.001$; this is consistent with past research finding that White respondents ($M=48.39$) overestimate Black-White wealth equality to a greater extent than do people of color ($M=37.12$). There was no three-way interaction $F(6,568)=0.35$, $p=.908$.

Speech differences by condition. A supplementary analysis comparing speech differences between the data/combined and narrative intervention conditions found a significant effect for achievement related words, aligning with our expectation that the data/combined

intervention would reduce the use of achievement related speech surrounding racial inequality (Table S1). No statistically significant differences emerged for sentence length, word length, or the negativity and positivity of words. Interestingly, the data/combined intervention conditions elicited more speech than the narrative condition, which could be indicative of respondents processing the data they were exposed to in that condition.

Table S1. LIWC analysis examining data/combined versus narrative intervention mean differences in speech at time 2. An asterisk indicates that p < .05.

LIWC Analysis	T value	Data/ Combined	Narrative
Achievement	2.50*	1.99	2.39
Word count	-2.58*	584.70	466.50
Words per sentence	-0.63	18.42	18.01
Six letter words or longer	0.41	14.91	15.08
Positive words	1.09	3.53	3.76
Negative words	1.08	0.80	0.90

Predicting attrition at time 3 and 4. We conducted two binary logistic regression analyses at time 3 and 4 examining attrition rates as a function of respondent estimates of Black-White wealth equality at time 1, gender, race, political ideology, age, education, and income. In the time 3 analysis, no measures were significant predictors of returning to the experiment, though when relaxing the criteria for statistical significance to $p < .10$, higher income B=.103, SE = 0.059, $Wald$ = 3.037, p = .081, and more liberal B=-.184, SE = .095, $Wald$ = 3.753, p = .053 respondents tended to return survey responses. At time 4, only gender was a significant predictor of attrition B=-.566, SE = .262, $Wald$ = 4.680, p = .031, with women returning to the experiment more than men. When we relax the criteria for statistical significance to $p < .10$, higher income B=.107, SE = .060, $Wald$ = 3.177, p = .075, White racial identity B= .270, SE = .139, $Wald$ = 3.748, p = .053, and liberal ideology B=-.162, SE = .096, $Wald$ = 2.854, p = .091 predicted returning survey responses at time 4. No other variables significantly predicted attrition at time 3 and 4 ($Wald$ < 1.36, p > .245). A separate binary logististic regression analysis comparing the

data conditions to the narrative condition revealed no evidence of differential attrition by intervention type at time 3 (*Wald* = 1.223, *p* = .269) or time 4 (*Wald* = 0.118, *p* = .731). Overall, this analysis indicates that attirtion was unsystematic across our sample's demographic characteristics and general political attitudes.

Inverse probability weighting. Though no systematic prediction of attrition was observed in our study, there are both practical and psychological reasons why extreme responses on our pretest assessment of respondent beliefs about Black-White wealth equality might explain attrition in our follow-up study. For practical reasons, those who make extreme scores might have a harder time understanding the math in our survey responses and might select out of the survey. For psychological reasons, extreme scores on the perception of racial wealth inequality mean that our interventions might be challenging respondent beliefs most and thus leading to more attrition. We conducted an inverse probability weighting analysis based on these scores to account for this tendency (4). We conducted this analysis for both the time 3 and time 4 follow-up responses, in both cases weighting responses by their baseline estimates for Black-White wealth equality. For both time points we find results that are consistent with those reported in the main text (see Table S2).

Table S2. Linear regression predicting Black-White wealth inequality estimates at Time 3 and 4 as a function of intervention collapsed across the structural/combined conditions and control variables using inverse probability weighting on Time 1 responses of Black-White wealth inequality. An asterisk indicates that p < .05, and a plus sign indicates p < .10.

Variable	B (SE)<br>Time 3	B (SE)<br>Time 4
(Constant)	33.28 (9.64)*	18.47 (9.94)$^{+}$
Black-White wealth equality (time 1)	0.67 (0.05)*	0.31 (0.06)*
Structural/combined intevention	-15.02 (3.26)*	-8.36 (3.49)*
Race	2.20 (1.76)	2.92 (1.87)
Gender	1.05 (3.43)	2.03 (3.79)
Income	0.48 (0.76)	1.52 (0.80)$^{+}$
Ideology	1.56 (1.24)	3.82 (1.26)*
Education	-1.37 (1.76)	-3.28 (1.95)
Age	-0.04 (0.14)	0.04 (0.15)

Moderation by SDO and political ideology. Theoretically, our manipulation is likely to be most effective for respondents who tend to be most liberal and in particular, most egalitarian, and by contrast the intervention will be least effective for those high in conservatism and in particular, social dominance (5-6). We tested this with two linear regression analyses predicting Black-White wealth equality estimates with the structural/combined intervention and its interaction with Social Dominance Orientation or political ideology. In the first analysis, we adjusted for time 1 estimates of Black-White wealth equality, race, income, education, ideology, gender, and age. The results of this analysis find some support for a reduced effectiveness of the intervention among high SDO respondents (see Table S3). As the Table shows, the structural/combined intervention again reduced time 2 estimates of Black-White wealth equality, and time 1 estimates were again a significant predictor of time 2. In this model a significant effect of SDO emerged such that high SDO respondents reported higher time 2 Black-White wealth equality. When relaxing criteria for statsitical significance to $p < .10$, the intervention and SDO effects were qualified by a significant interaction that was in line with the above expectations: the structural/combined intervention was less effective at moving respondents who were high versus low in SDO.

Table S3. Linear regression predicting Black-White wealth inequality estimates at Time 2 as a function of intervention collapsed across the structural/combined conditions and SDO, adjusting for controls. An asterisk indicates that p < .05, and a plus sign indicates p < .10.

Variable	B	SE	t
(Constant)	7.87	9.47	0.831
Black-White wealth equality (time 1)	0.39	0.04	8.938*
Structural/combined intevention	-16.03	7.38	-2.172*
SDO	8.50	2.60	3.272*
Race	2.65	1.41	1.878^{+}
Gender	1.66	2.81	0.591
Income	-1.18	0.62	-1.898^{+}
Ideology	0.28	1.10	0.255
Education	1.25	1.45	0.860

Age	0.049	0.12	0.412
Interaction	-5.56	2.92	-1.907+

Though there was some suggestive evidence of an interaction between the intervention and SDO, there was no such interaction for ideology. This second linear regression analysis is presented in Table S4. In this analysis only the intervention, time 1 estimates of Black-White wealth equality, and SDO emerged as significant predictors.

Table S4. Linear regression predicting Black-White wealth inequality estimates at Time 2 as a function of intervention collapsed across the structural/combined conditions and ideology, adjusting for controls. An asterisk indicates that $p < .05$, and a plus sign indicates $p < .10$.

Variable	B	SE	t
(Constant)	16.98	9.11	1.864+
Black-White wealth equality (time 1)	0.39	0.04	8.856*
Structural/combined intevention	-29.50	6.74	-4.374*
Ideology	0.43	1.76	0.245
Race	2.70	1.42	1.901+
Gender	1.86	2.82	0.658
Income	-1.11	0.62	-1.778+
SDO	4.60	1.62	2.849*
Education	1.25	1.46	0.858
Age	0.03	0.12	0.264
Interaction	0.16	2.00	0.081

Moderation by age. We contend that interventions such as this should be conducted as early and as often as possible. Nevertheless, one reasonable hypothesis given the results of our study is that an educational intervention, like the one we use in this study, would be best suited to younger audiences with less deeply entrenched beliefs about racial inequality. However, we believe there are at least a few reasons to be skeptical of this hypothesis. According to a large and growing body of research, racial socialization processes happen at very early ages in American families (7). As a result of this socialization process, entrenched beliefs about racial inequality are likely to be present at the time when children are able to understand more complex

components of societal structures through data—such as those we used in our data interventions (8). As well, though children may have more malleable views about racial inequality, these views are also likely to be actively shaped by adults, as part of the curriculum of primary education institutions, who are likely to adhere to narratives of racial progress in their pedagogical choices (9-10).

Analysis of our own data, capitalizing on the age range of our adult sample, also suggests caution about the heightened effectiveness of this sort of intervention with younger populations: We conducted three linear regression analyses examining the extent that age moderated the effect of the intervention on respondent estimates of the Black-White wealth gap. None of these regression analyses found a significant effect of age ($ts < 1.38$, $ps > .168$) or a statistically significant interaction between the intervention and age ($ts < 1.53$, $ps > .128$). Importantly, because these analyses are with adults, these data cannot directly answer the question of the differential effectiveness of the intervention among children.

Pandemic questionnaires and descriptive statistics. At time 4 respondents answered questions about their experiences during the pandemic caused by the proliferation and lack of containment, due to government negligence and malpractice, of COVID-19. Of our time 4 respondents, several had their work impacted by the pandemic: 22.46% had lost jobs, 24.64% had changed jobs, 38.65% had their jobs cut back/restricted, 5.31% had their company go out of business, and 85.54% had their work/school closed during the pandemic. Several also had negative health outcomes: 5.31% had tested postive for COVID-19, 15.46% became sick, and 6.28% were hospitalized. Our respondents were also active in politics during this time, with 40.10% joining a anti-police violence of Black Lives Matter protest, and 2.90% joining a reopen the economy protest.

Respondents also estimated the amount of positive cases and deaths of Black people due to COVID-19 for every 100 White people on the same 0 to 200 slider scale as our wealth gap items. Respondents estimated that Black people tested positive at a rate of 142.46 to 100 and died at a rate of 148.12 to 100 per White people due to COVID-19. Estimates of these case and death statistics continue to vary but most find that infection and death rates exceed 2 to 1 (https://www.cdc.gov/coronavirus/2019-ncov/community/health-equity) for Black versus White communities, making the responses generated by our respondents overestimates of the prevalence of COVID-19 Black-White equality. This pattern is consistent with our observed wealth gap estimates.

Supplementary References

1. Kiatpongsan, S., & Norton, M. I. (2014). How much (more) should CEOs make? A universal desire for more equal pay. *Perspectives on Psychological Science*, *9*(6), 587-593.
2. Day, M. V., & Fiske, S. T. (2017). Movin'on up? How perceptions of social mobility affect our willingness to defend the system. *Social Psychological and Personality Science*, *8*(3), 267-274.
3. Ho, A. K., Sidanius, J., Kteily, N., Sheehy-Skeffington, J., Pratto, F., Henkel, K. E., ... & Stewart, A. L. (2015). The nature of social dominance orientation: Theorizing and measuring preferences for intergroup inequality using the new SDO_7 scale. *Journal of Personality and Social Psychology*, *109*(6), 1003.
4. Gomila, R., & Clark, C. S. (2020). Missing data in experiments: Challenges and solutions. *Psychological Methods*.
5. Richeson, J. A., & Sommers, S. R. (2016). Toward a social psychology of race and race relations for the twenty-first century. *Annual review of psychology*, *67*, 439-463.

6. Sidanius, J., & Pratto, F. (2001). *Social dominance: An intergroup theory of social hierarchy and oppression*. Cambridge University Press.
7. Abaied, J. L., & Perry, S. P. (2021). Socialization of racial ideology by White parents. *Cultural diversity and ethnic minority psychology*.
8. Lavigne, N. C., & Lajoie, S. P. (2007). Statistical reasoning of middle school children engaged in survey inquiry. *Contemporary Educational Psychology*, *32*(4), 630-666.
9. Nelson, J. C., Adams, G., & Salter, P. S. (2013). The Marley hypothesis: Denial of racism reflects ignorance of history. *Psychological science*, *24*(2), 213-218.
10. Seamster, L., & Ray, V. (2018). Against teleology in the study of race: Toward the abolition of the progress paradigm. *Sociological Theory*, *36*(4), 315-342.
